# Experiences of Malaysian primary healthcare providers with vaccine hesitancy: A qualitative study

**DOI:** 10.51866/oa.388

**Published:** 2024-04-14

**Authors:** Megat Mohamad Amirul Amzar Megat Hashim, Nik Sherina Haidi Hanafi

**Affiliations:** 1 MBBS, MMed, Department of Primary Care Medicine, University of Malaya Medical Centre, 59100, Kuala Lumpur, Malaysia. Email: amirul.amzar@ummc.edu.my; 2 MBBS, MMed, PhD, Department of Primary Care Medicine, Faculty of Medicine, University of Malaya, 50603, Kuala Lumpur, Malaysia.

**Keywords:** Vaccine, Vaccine hesitancy, Primary healthcare provider, Vaccine refusal, Antivaccine

## Abstract

**Introduction:**

Vaccine hesitancy was declared as one of the ten threats to global public health by the World Health Organization in 2019. It undermines the effort towards eradication of vaccine-preventable diseases. Healthcare providers, who are directly involved in vaccination services and vaccine advocacies, are important in combating vaccine hesitancy. Studies have shown that vaccine refusers have various reasons for refusal including distrust towards healthcare providers. Hence, it is important to understand healthcare providers’ perspectives. This study aimed to explore primary healthcare providers (PHCPs)’ experiences in dealing with vaccine hesitancy.

**Methods:**

This qualitative study was conducted among public PHCPs across six states in Malaysia. Purposive and snowball sampling methods were used. Fifteen primary healthcare doctors and nurses underwent in-depth interviews. Recruitment was stopped when data saturation was achieved. Data were thematically analysed.

**Results:**

Four themes emerged: 1) views towards vaccination and vaccine hesitancy, 2) disparity in strategies and resources used among PHCPs, 3) fixed-minded vaccine deniers and religious incompatibility: the two towering hurdles and 4) negative impact after encounters with vaccine hesitancy.

**Conclusion:**

Malaysian PHCPs encounter negative experiences with vaccine hesitancy, impacting them negatively. These experiences are attributed to the challenges and lack of standardised resources for reference. These findings suggest the development of a more flexible policy, a training module inclusive of all professional roles and a standardised repository of resources for managing vaccine hesitancy.

## Introduction

Vaccine hesitancy, defined as the reluctance or refusal to receive vaccination despite the availability of vaccines, was declared by the World Health Organization (WHO) as one of the threats to global public health in 2019.^1,2^ It is a barrier to the eradication of vaccine-preventable diseases. The progress towards the Global Vaccine Action Plan target of at least 90% coverage for all assessed vaccines in 2019 is stalled or even reversed in certain countries.^3^ As such, the incidence of vaccine-preventable diseases due to vaccine hesitancy is increasing and is especially alarming in Muslim countries including Malaysia.^4^ Locally, vaccine hesitancy has led to an increase in vaccine-preventable diseases and the emergence of eradicated diseases such as poliomyelitis.^5^ As confidence towards vaccination includes confidence towards service providers, distrust towards healthcare providers is one of the reasons for vaccine refusal.^6^ This is perturbing, as the WHO views healthcare providers, especially those working in the community, as the influencers of vaccination decisions.^1^

Malaysia is a developing, multi-ethnic and multireligious country in Southeast Asia. The majority of the population in the country is Muslim. Religious incompatibility has been described as another factor influencing vaccine acceptance.^7^ As Malaysian primary healthcare providers (PHCPs) may have different religions to their client, understanding the dynamic of this relationship is important. In general, Malaysian childhood vaccination service is provided mostly by the public health facilities, subsidised by the government^8^. PHCP in public health clinics are the main vaccine service providers. The current local guideline highlights the referral system should the clients refuse vaccination.^9^ The first point of counselling starts with nurses and escalates to medical officers and then to family medicine specialists (FMSs) should clients still refuse vaccination. This system has caused discomfort among parents, as they view repeated visits as coercion towards them.^10^

It is important to understand healthcare providers’ experiences with vaccine hesitancy in Malaysia and the impact of these experiences on them. These views will provide insights into the relationship between vaccine-hesitant parents and healthcare providers, ultimately aiding in the development of guidelines and strategies for addressing vaccine hesitancy.

## Methods

### Study design

This study utilised a qualitative approach. The integrated behavioural model (IBM) was used as the theoretical framework to understand PHCPs’ behaviour towards vaccine hesitancy. The constructs of the IBM were used to explore PHCPs’ experiences including the challenges encountered, strategies employed and impacts observed in dealing with vaccine hesitancy.^11^

### Study setting and participants

The study was conducted from March to October 2021 in public health clinics across six states in Malaysia whose incidence of vaccine refusal varied. The participants were PHCPs including FMSs, medical officers and nurses working in the selected clinics. The inclusion criteria were 1) at least one encounter with vaccine hesitancy in their practice and 2) the ability to converse in either English or Malay.

As per the Malaysian National Childhood Immunisation Programme, vaccination starts on the first day of life.^8^ After newborns are discharged, home visits are conducted by community nurses nine times up to 20 days of puerperium.^12^ This is usually the first contact of PHCPs with vaccine-hesitant parents. Community nurses then counsel parents and refer them to medical officers and then to FMSs should counselling fail. Occasionally, registered nurses and nursing sisters are involved prior to referral to medical officers. Nursing sisters have more training and experience than registered nurses and registered nurses than community nurses.

### Sampling and recruitment

Participants were recruited via purposive and snowball sampling methods. FMSs with known involvement in vaccine hesitancy advocacies were approached to participate and asked to suggest other participants. Advice from state health departments about clinics with high vaccine hesitancy rates was also sought.

### Data collection

Data were collected via in-depth interviews (IDIs) to encourage active participation and expression of opinion without influence and pressure from differences in hierarchy. All interviews were conducted by the principal researcher. In view of the COVID-19 pandemic, the IDIs were conducted via teleconferencing using a secure audio-visual platform (i.e. Zoom), which was encrypted to ensure data security.^13^ The interviews were conducted in the preferred language of participants and recorded using an audio recorder. Field notes were taken to aid analysis. Sampling was stopped after data saturation was attained.^14^

### Data analysis

The audio recordings were transcribed verbatim, coded and analysed in their original languages. Malay words or sentences were translated into English by the researchers for reporting purposes. Content analysis was conducted with the aim of developing themes.^15^ The two researchers analysed the transcripts independently and then discussed and agreed on the coding framework. Thereafter, the code groups were organised into a list of themes that were produced as the final results of the data analysis.

Several strategies were employed to ensure research rigour. Triangulation was conducted by obtaining data from various professional positions and social backgrounds. A log of the research process was kept to ensure an audit trail. The decision on the methodology and interpretive judgement of the data analysis were noted with their justifications. A final discussion was carried out between the researchers regarding the list of themes to ensure a neutral interpretation and prevent researcher bias.

## Results

### Participant demographics

A total of 15 participants (five FMSs, four medical officers, five nurses and one nursing sister) were interviewed. Their ages ranged from 31 to 53 years. Thirteen participants were Muslims, while the remaining two were Hindus (Table 1). The participants were recruited from seven public health clinics across six states: Johor (n=5), Sabah (n=3), Terengganu (n=3), Kedah (n=2), Kuala Lumpur (n=1) and Selangor (n=1).

**Table 1:** Participant demographics.

No.	Age (year)	Sex	Religion	Occupation
P1	33	Male	Muslim	Medical officer
P2	40	Female	Muslim	Family medicine specialist
P3	46	Male	Hindu	Family medicine specialist
P4	50	Male	Muslim	Family medicine specialist
P5	45	Female	Muslim	Family medicine specialist
P6	31	Female	Muslim	Community nurse
P7	33	Female	Muslim	Medical officer
P8	46	Female	Muslim	Community nurse
P9	41	Female	Muslim	Community nurse
P10	34	Female	Muslim	Medical officer
P11	42	Female	Hindu	Family medicine specialist
P12	39	Female	Muslim	Nurse
P13	53	Female	Muslim	Nursing sister
P14	49	Female	Muslim	Community nurse
P15	34	Female	Muslim	Medical officer

### Themes

The themes, subthemes and representative quotes are summarised in Table 2.

**Table 2:** Themes, subthemes and representative quotes.

Theme	Subtheme	Representative quote
**Theme 1:** Views towards vaccination and vaccine hesitancy	Confidence in the benefit and safety of vaccination	*‘I think vaccination, especially among kids, has been proven to save lots of lives’.* – P5 *‘...If we got the disease, it is much worse than if we got side effects from the vaccine’.* – P7
	Negative perception towards vaccine hesitancy	*“I felt like he is an irresponsible father, who do not think about his children's future*– P6
**Theme 2:** Disparity in strategies and resources used among PHCPs	Higher-ranking PHCPs receiving regular training	*‘Usually, we get information regarding vaccines from CME (continuous medical education) because we will have CME every year regarding vaccines’.* – P7, a medical officer
	Nurses having a poor self-perception of their effort and knowledge in managing vaccine hesitancy	*‘...That time I feel so weak with the position and knowledge that we have. What we told them was something we knew, studied and practised. But when they did not listen and react, I felt disappointed. So we need to refer...’.* – P9, a community nurse
	Disparity in resources used for reference	*‘...I gather information online. We do have a few good speakers like Dr MN, Dr S, and we do have some portals like Immunize For Life, and we do have certain books even by Dr S and others...* – P11, an FMS *‘...Because I am just a community nurse, I just deliver what I have been taught. For a more detailed explanation, I have to use Google and learn from the internet’...* – P9, a community nurse
**Theme 3:** Fixed-minded vaccine deniers and religious incompatibility: the two towering hurdles	Non-cooperation from fixed-minded vaccine refusers	*“We were chased out! Even before we got to enter their house. They won’t let us, they said no need to explain”.* – P8
	PHCPs being forced to deal with fixed-minded vaccine refusers repeatedly	*‘.The previous FMS, he pressured us too much. That time all the staff were stressed. The (nursing) sister and matron had to follow. We had to go (home visit) for as long as the mother refuse to come.* – P8
	Low rate of success despite maximal effort and time spent	*I spend at least about 1 hour with them, none has agreed for vaccine’.* – P3 *“It will take a lot of time in doing so, because you are actually giving them all the facts. But in the end, they still didn't appreciate the facts”.* – P5
		*“...He (the vaccine refuser) said to me that there was non-permissible content, like porcine gelatin. He said, due to that, he doesn't want to. He said it is non-permissible for his children’s body*– P8, a Muslim community nurse managing a Muslim client
	Religion being used to refuse vaccination	*“They hide behind the religion, they think you don't know... One patient told me that she followed the methods during the prophet’s time of preventing all the diseases. I don’t have much knowledge on that. So I cannot defend. So they shut you out by saying, “ohh... it’s a part of my religion". We can’t say anything".* – P3, an Indian FMS managing a Muslim client
	Sadness and frustration lingering after encountering vaccine hesitancy	*‘We felt sad. We felt like that because we went by car, by motorcycle to their house, on rainy days, on sunny days, we still went. After that, when we arrived, they chased us out as if we are just a stray dog’.* – P8
**Theme 4:** Negative impact after encounters with vaccine hesitancy	Decreasing morale due to a low rate of success despite maximal effort	*It really brings our morale down among the staff, because we think that we have tried so many things, we have actually given the information, we try to actually talk to them, but with little result, or I would say no result at all’.* – P5 *“It’s like we have failed, as a nurse I failed to convince him to get the vaccine”.* – P12
	Negative emotions affecting the professional practice of PHCPs	*“That’s what made us feel challenged. And then, sometimes it leads to us raising our voice to the patient. Sometimes we didn't even realise it”.* – P7

FMS, family medicine specialist; PHCP, primary healthcare provider

### Theme 1: Views towards vaccination and vaccine hesitancy

The interviewed PHCPs expressed high confidence in the benefit and safety of vaccination. Consequently, they shared negative perceptions towards individuals who refuse vaccination. They perceived vaccine refusal as an irresponsible choice, as it may affect the community, hence leading to negative emotions in dealing with vaccine hesitancy.

### Theme 2: Disparity in strategies and resources used among PHCPs

The PHCPs reported no standardised method for handling vaccine hesitancy. The doctors received regular training and tended to be more equipped in terms of reference and skills. In contrast, the majority of the nurses were not trained and used non-credible resources as reference. This led to their self-perceived inferiority in dealing with vaccine hesitancy despite being the first to encounter it.

### Theme 3: Fixed-minded vaccine deniers and religious incompatibility: the two towering hurdles

The PHCPs shared that fixed-minded vaccine refusers are often uncooperative. The PHCPs were forced to deal with them repeatedly due to the rigid standard operating procedure (SOP). Despite maximal effort and time spent, it was difficult to persuade fixed-minded refusers. Additionally, religion was reported by the PHCPs as being used often by their clients in refusing vaccination, which became more profound when the counselling PHCPs came from a different religion.

### Theme 4: Negative impact after encounters with vaccine hesitancy

The negative perception towards vaccine hesitancy, propagated by the repeated negative experiences surrounding their encounters, led to frustration among the PHCPs. This perception was worsened by the low rate of success, leading to reductions in the morale and negative effects on the professional practice of the PHCPs.

## Discussion

Vaccine hesitancy is a spectrum. Fixed-minded vaccine refusers encountered by PHCPs are likely to be vaccine deniers. The group at the end of the vaccine hesitancy spectrum has a substantially negative attitude towards vaccination and is not willing to change despite scientific explanations.^16^ Encountering these clients causes internal conflicts when PHCPs try to balance their obligations towards individual decisions and the necessity to prevent communicable disease transmission in the community.^17^ This leads to frustrations, which become more prominent among encounters with parents who are unwilling to even engage in discussions.

Religious concerns are one of the reasons for vaccine refusal.^10,18^ This reason emerges even among PHCPs and clients who share the same religion, and it becomes more prominent when they do not. PHCPs may respond to this through various manners, most notably by providing more medical information and discussing the decision-making process.^19^ However, as religion affects clinical practice,^20^ religious disparity may not only cause discomfort among PHCPs and clients but also lead to suboptimal attempts of communication regarding vaccination. Without intervention, this aspect may deepen the misunderstanding of religious incompatibility, especially in multireligious countries such as Malaysia.

Choosing to dismiss vaccine deniers and focusing only on fence-sitters were not possible for the participants in this study. As PHCPs are working in public healthcare facilities, they are required to adhere to the rigid SOP for vaccine hesitancy.^9^ Due to the hierarchical escalation system, vaccine refusers will not be dismissed until maximal effort is given to convince them. This may be different with healthcare providers working in the private sector, as it has been shown that they are more likely to dismiss vaccine-refusing clients.^21^ Conversely, the referral system benefits vaccine-hesitant clients by ensuring that they have maximal information and explanation to make an informed decision. However, this approach puts both clients and staff in discomfort due to repeated stressful negative encounters.

The resources used were not standardised and differed greatly among the nurses and doctors who participated in the study. At the time of the study, there were no specific guidelines or a standardised information repository for communicating with vaccine refusers in Malaysia. Although the doctors used credible resources, it was worrying that the nurses used Google and YouTube to assist them, as the usage of different keywords may lead to false information regarding vaccination.^22^ More than half of internet users have been reported to perceive vaccination-related information obtained from the internet as accurate, even though it is incorrect half of the time.^23^ Hence, by not having standardised sources of credible information, nurses may provide inaccurate information to vaccine-hesitant parents and pose more challenges for future consultations.

All of the abovementioned factors generally result in a negative experience among PHCPs in dealing with vaccine hesitancy. The perceived benefit that vaccination prevents diseases and outbreaks and confers herd immunity^24^ is challenged when they encounter vaccine hesitancy. This negative experience has a significant impact. The feelings reported by PHCPs fit the three components of burnout syndrome.^25^ Being scolded by clients is one of the significant contributing factors to burnout.^26^ Loss of autonomy at work and decreased control over the work environment, wherein nurses are coerced to meet vaccine refusers repeatedly, add to the negative impact noted.^27^ Consequently, the negative impact affects their practice, possibly leading to medical error and a decline in patient safety.^28^

This study is limited by the variability of the participants. Out of 2863 public health clinics in Malaysia, only seven were included.^29^ Private practitioners were not interviewed, and only two religions were represented. Nevertheless, as this study focused on understanding the experiences and challenges in vaccine hesitancy counselling, the source triangulation among the PHCPs of varying professional positions and social backgrounds provided rich insights.

In conclusion, Malaysian PHCPs mostly have negative experiences with vaccine hesitancy particularly in dealing with fixed-minded vaccine refusers, worsened by the rigid existing policy and religious disparity. Actions should be taken to revise the current hierarchical escalation system, especially in dealing with different spectrums of vaccine hesitancy. In addition, a standardised training module and a vaccination information repository should be developed to prepare PHCPs, including both doctors and nurses, to manage vaccine hesitancy. Cooperation with religious authorities and individuals should be sought to manage the religious concerns related to vaccine hesitancy in multireligious countries such as Malaysia. Lastly, further studies comparing the knowledge, attitude and confidence towards vaccines between providers and clients should be conducted to understand the impact of any disparity.

## Data sharing statement

Raw data are available upon request.

**How does this paper make a difference in general practice?**

- This study provides insights into the negative experiences of public primary healthcare providers in Malaysia in dealing with vaccine hesitancy.
- The rigid existing policy causes repetitive encounters of primary healthcare providers with fixed-minded vaccine deniers and impairs the relationship between both parties.
- The lack of standardised resources causes disparity in managing vaccine hesitancy among public healthcare providers.
- The religious disparity that occurs even within the same religion causes communication roadblocks in vaccine hesitancy counselling.
- Understanding the abovementioned factors can help in designing a more pragmatic policy that is more understanding towards both providers and clients.
